# Gut sensing of food ingredients and interoception‐mediated regulation of feeding and glucose metabolism

**DOI:** 10.14814/phy2.70636

**Published:** 2025-10-27

**Authors:** Kengo Iba, Rika Kitano, Yusaku Iwasaki

**Affiliations:** ^1^ Laboratory of Animal Functional Science, Graduate School of Life and Environmental Sciences Kyoto Prefectural University Kyoto Japan

**Keywords:** gastrointestinal distention, interoception, meal‐related physiology, postprandial hormones, vagal afferent nerves

## Abstract

Food intake not only provides pleasure through exteroceptive sensations such as taste and smell but also elicits beneficial physiological effects via interoceptive signals arising from the gastrointestinal tract and beyond. Among these interoceptive pathways, vagal sensory (vagal afferent) nerves play a central role in transmitting food‐derived information to the brain. This review first outlines the anatomical and functional characteristics of vagal sensory nerves. It then examines how food‐related signals, including mechanical stretching of the gastrointestinal wall, gastrointestinal and pancreatic hormones such as glucagon‐like peptide‐1, and microbial metabolites like short‐chain fatty acids, are detected by vagal pathways. These inputs collectively regulate food intake, nutrient preferences, and systemic metabolism. Recent studies further suggest that vagal sensory nerves enable the brain to anticipate and adapt to the metabolic demands of food intake, serving as a key mechanism for maintaining homeostasis during rapid postprandial changes. Understanding the role of vagal afferents in sensing meal‐derived signals and mediating gut–brain communication provides insights into how interoceptive pathways orchestrate energy balance and hold promise for developing therapeutic strategies for metabolic disorders such as obesity and diabetes.

## 1 INTRODUCTION

Animals, including humans, obtain all the energy necessary to sustain life from food. Eating is thus a fundamental biological activity that underpins survival. Beyond its metabolic role, eating also contributes to emotional well‐being by providing enjoyment and a sense of satisfaction. In recent years, anti‐obesity drugs such as glucagon‐like peptide‐1 receptor agonists have been developed to suppress appetite and promote weight loss. However, these medications may also unintentionally diminish the psychological pleasure associated with eating by blunting hunger and food cravings.

Through exteroceptive sensory modalities such as smell, taste, and texture, we evaluate the safety and nutritional value of food while also experiencing its palatability and rewarding qualities. In parallel, growing evidence indicates that food ingestion also activates interoceptive pathways: mechanical and chemical stimuli generated as food passes through the gastrointestinal (GI) tract are transmitted subconsciously to the brain. These signals modulate key meal‐related physiological responses, including appetite regulation, reward processing, and glucose metabolism in anticipation of nutrient absorption. Central to this interoceptive sensing are vagal sensory nerves, which convey visceral information from the gut to the brain. This review summarizes current advances in our understanding of how vagal sensory pathways detect food‐derived signals and how these signals shape both central and systemic physiological functions.

## 2 VAGAL SENSORY NERVES AND THEIR ANATOMICAL DISTRIBUTION

Many organisms, including humans, possess two principal sensory modalities: exteroception, which detects stimuli from the external environment, and interoception, which monitors internal physiological states. Exteroception encompasses sensory inputs such as vision, hearing, smell, taste, and touch, all of which contribute to the conscious perception of the outside world. In contrast, interoception involves the subconscious detection of internal bodily signals, such as gastrointestinal distension, blood glucose levels, blood pressure, heart rate, and respiratory dynamics. These interoceptive signals are relayed to the brain without entering conscious awareness. Feeding behavior is traditionally guided by exteroceptive cues—such as the taste, smell, and texture of food—which influence food preferences and palatability. However, recent studies have shown that interoceptive signals also play a critical role, not only in the generation of satiation and satiety, but also in shaping nutrient‐specific reward and food preference.

The vagal afferent (or vagal sensory) nerves are the primary conduit for conveying interoceptive signals from the viscera to the brain. The vagus nerve, the tenth cranial nerve (CN X), arises from the medulla and projects extensively to the head, thorax, and abdominal organs. Although often associated with parasympathetic efferent output, approximately 75%–90% of vagal fibers are afferent (Paintal, 1963; Prechtl & Powley, 1990), transmitting sensory information from the viscera to the brain.

**FIGURE 1:**
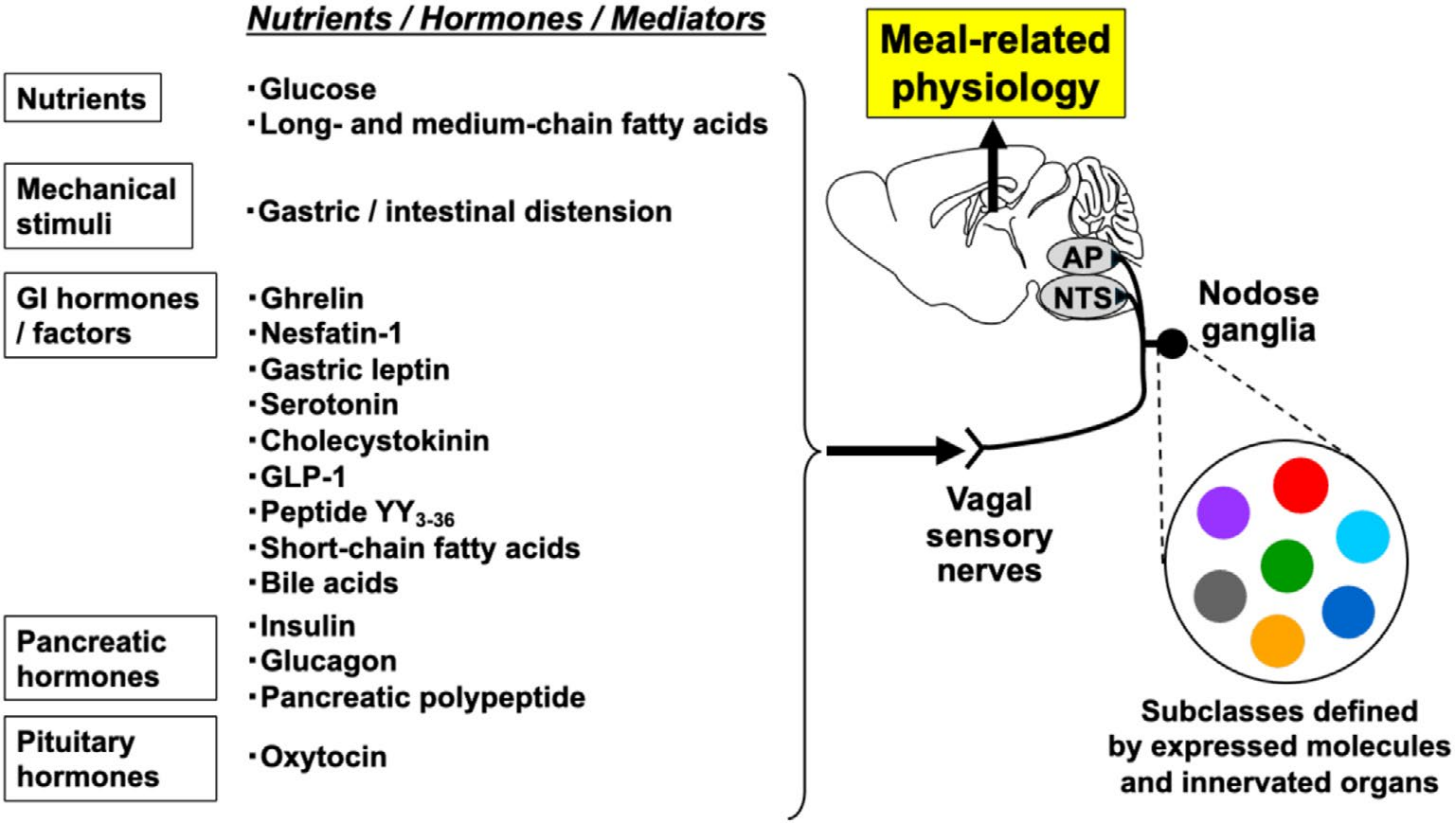
Schematic overview of meal‐related factors acting on vagal sensory nerves and their links to meal‐related physiology. Meal‐related factors (nutrients, hormones, and mediators) directly act on vagal sensory nerves, and the involved receptors are listed in Table 1. Vagal sensory neurons comprise multiple subclasses defined by their receptor and molecular expression patterns and by the peripheral organs they innervate (Bai et al., 2019; Kupari et al., 2019). Distinctly colored circles in the schematic represent these different subclasses of vagal sensory neurons. AP, area postrema; NTS, nucleus tractus solitarius.

Vagal sensory neurons are pseudounipolar neurons whose cell bodies are located in the jugular and nodose ganglia near the jugular foramen. Each neuron extends a single axon that bifurcates to project peripherally toward visceral and somatic targets, and centrally into the brainstem. Peripherally, vagal axons travel within the carotid sheath, innervating diverse structures including the meninges, auricle, heart, lung, trachea, larynx, esophagus, stomach, intestine, liver, pancreas, thyroid, and arteries. Within these tissues, vagal terminals exhibit morphological and molecular diversity, presumably enabling detection of distinct sensory cues. Jugular ganglion neurons predominantly innervate somatic and proximal visceral structures such as the auricle, meninges, larynx, and respiratory tract, and project centrally to the paratrigeminal nucleus (Pa5), a subnucleus within the spinal trigeminal tract (Caous et al., 2001; Mazzone & Undem, 2016). These neurons primarily function in exteroception and nociception. In contrast, nodose ganglion neurons project peripherally to thoracic and abdominal viscera and centrally to the caudal nucleus tractus solitarius (NTS) and area postrema (AP), where they mediate interoceptive signals such as visceral stretch, chemical cues, and internal organ state (Berthoud et al., 2025; Prescott & Liberles, 2022; Wang et al., 2020) (Figure 1). The NTS, the principal target of vagal sensory inputs, projects broadly throughout the central nervous system, including the brainstem, spinal cord, pons and midbrain, diencephalon (hypothalamus and thalamus), and forebrain, thereby exerting diverse physiological functions (Aklan et al., 2020). Several molecularly defined neuronal subclasses within the NTS, including those expressing *Th*; tyrosine hydroxylase (Aklan et al., 2020; Date et al., 2006), *Cck*; cholecystokinin (Roman et al., 2017), *Gcg*; glucagon (Gaykema et al., 2017; Holt et al., 2019), *Lepr*; leptin receptor (Cheng, Ndoka, et al., 2020), *Pomc*; proopiomelanocortin (Zhan et al., 2013), *Calcr*; calcitonin receptor (Cheng, Gonzalez, et al., 2020), *Prlh*; prolactin releasing hormone (PrRP) (Cheng et al., 2021), and *Adcyap*; adenylate cyclase activating polypeptide or pituitary adenylate cyclase‐activating polypeptide (PACAP) (Ilanges et al., 2022), have been implicated in the regulation of food intake. Projections from the NTS to the arcuate nucleus (ARC) of the hypothalamus promote feeding behavior either by activating agouti‐related peptide (AgRP) neurons or by inhibiting POMC neurons (Aklan et al., 2020; Date et al., 2006). In contrast, intestinal distension induced by hypertonic solutions activates oxytocin receptor‐expressing vagal sensory neurons, and the resulting neural signals ultimately suppress AgRP neuronal activity in the ARC, thereby inducing short‐term suppression of food intake (satiation) (Bai et al., 2019). NTS projections to the parabrachial nucleus (PBN) in the pons are also involved in feeding suppression. Interestingly, projections from CCK‐expressing NTS neurons to CGRP neurons in the PBN elicit aversive responses, whereas projections from Calcr‐expressing NTS neurons to non‐CGRP PBN neurons reduce food intake without inducing aversion (Cheng, Ndoka, et al., 2020; Roman et al., 2017). Moreover, mechanical stimulation caused by gastric distension activates prodynorphin‐expressing neurons in the PBN via the NTS, leading to a reduction in food intake accompanied by aversive responses (Kim et al., 2020).

**FIGURE 2:**
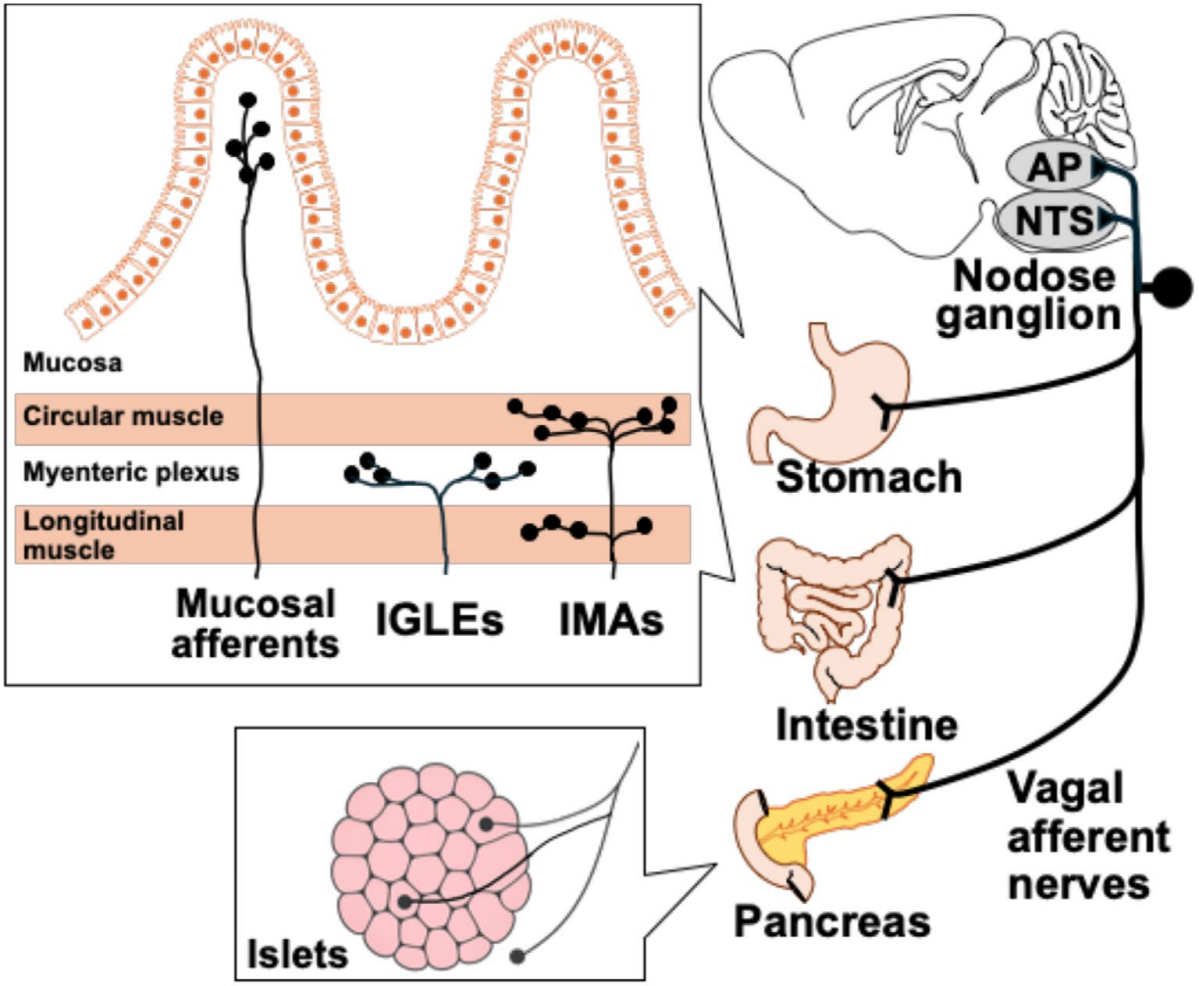
Vagal afferent nerves linking the gastrointestinal tract to the brain. Vagal nerve terminals innervating the gastrointestinal tract are distributed within the mucosa (mucosal afferents), the myenteric plexus between muscle layers (intraganglionic laminar endings; IGLEs), the longitudinal and circular muscle layers (intramuscular arrays; IMAs), and around or within pancreatic islets (Makhmutova et al., 2021). These afferent nerves detect signals derived from food or food‐evoked responses in the gut and the pancreas, which are transmitted to the nerve endings either via paracrine or synapse‐like mechanisms, and relay this neural information to the nucleus tractus solitarius (NTS) and area postrema (AP) in the medulla.

Vagal sensory innervation in the gastrointestinal (GI) tract comprises three major types of terminal specializations (Figure 2) (Berthoud et al., 2025; Prescott & Liberles, 2022; Wang et al., 2020). (1) Mucosal endings, located within the lamina propria of the intestinal mucosa, are responsible for detecting chemical stimuli such as nutrients and gut hormones. The other two types include (2) intraganglionic laminar endings (IGLEs), which are situated in the myenteric plexus, and intramuscular arrays (IMAs), which are found within the circular or longitudinal muscle layers. Both of these respond primarily to mechanical stimuli including distension of the gut wall.

Anatomical studies have shown that a subset of mucosal vagal afferent endings forms synapse‐like contacts with enteroendocrine cells in the intestinal epithelium. These specialized cells have been termed “neuropod cells” due to their neuronal properties and connectivity with sensory fibers (Bohorquez et al., 2015). However, such direct contacts appear to be relatively infrequent. The majority of mucosal endings are instead positioned adjacent to enteroendocrine cells, without forming distinct synaptic structures, at least in the case of enteroendocrine L cells that produce glucagon‐like peptide‐1 (GLP‐1) and peptide YY (PYY) (Cao et al., 2024). Thus, mucosal afferent endings are anatomically positioned to detect high concentrations of gut hormones immediately after secretion. This is likely accomplished through paracrine signaling, and in certain cases, through synapse‐like communication, enabling rapid and specific chemosensory detection within the intestinal environment.

Importantly, emerging studies using retrograde tracers reveal that individual vagal sensory neurons typically innervate only one target organ, rather than multiple visceral regions, reinforcing the concept of organ‐specific afferent signaling (Bai et al., 2019; Han et al., 2018; Williams et al., 2016; Zhao et al., 2022).

## 3 DIETARY FACTORS ACTING ON VAGAL SENSORY NERVES AND THEIR PHYSIOLOGICAL ROLES

Stimuli that activate vagal sensory nerves can be broadly classified into two categories: mechanical stimuli and chemical stimuli (Figure 1 and Table 1). Mechanical stimuli primarily include gastrointestinal distension and tension, whereas chemical stimuli encompass ingested nutrients, gastrointestinal and pancreatic hormones whose circulating levels fluctuate before and after meals, and microbial metabolites such as short‐chain fatty acids (SCFAs) produced by the gut microbiota. These stimuli do not act in isolation; rather, they often interact synergistically to modulate the activity of vagal sensory neurons. Through this modulation, they contribute to a wide range of postprandial physiological responses, including the induction of satiation and satiety, activation of reward‐related pathways, and regulation of glucose and energy metabolism.

**TABLE 1:** Meal‐related factors acting on vagal afferent nerves (VANs) and their roles in meal‐related physiology, particularly in the regulation of feeding.

Nutrients/hormones/mediators	Source	Receptors in VANs	Effect on VAN	Effect mediated via VAN	References
Nutrients					
Glucose (sugar)	Dietary origin	KATP channel, SGLT1	Activation / Inhibition	Sweet preference, feeding regulation?	Buchanan et al. (2022); Grabauskas et al. (2010); Tan et al. (2020); Zhou et al. (2011)
Long‐ and medium‐chain fatty acids	Dietary origin	GPR40 (FFAR1), GPR120 (FFAR4)	Activation / Inhibition	Fat preference, feeding regulation?	Darling et al. (2014); Li et al. (2022)
Mechanical stimuli					
Pectin‐containing carbonated water		?	Activation	Suppression of food intake	Ohbayashi et al. (2021)
GI hormones/factors					
Ghrelin	Gastric X/A‐like cells, P/D1 cells	Growth hormone secretagogue receptor	Inhibition	Stimulation of food intake	Date et al. (2002)
Nesfatin‐1	Gastric X/A‐like cells, Pancreatic β cells and others	Not identified	Activation	Suppression of food intake	Iwasaki et al. (2009)
Gastric leptin	Gastric chief cells, enteroendocrine P cells	Leptin receptor	Activation	Suppression of food intake	Peters et al. (2004, 2005)
Serotonin	Enterochromaffin cells	5‐HT_3_ receptor	Activation	Inhibition of gastric emptying, stimulation of exocrine pancreas secretion	Malone et al. (1991); Nawrot‐Porabka et al. (2013); Raybould (2010); Raybould et al. (2003)
Cholecystokinin	Enteroendocrine I cells	CCK‐A receptor	Activation	Suppression of food intake	Lankisch et al. (2002); Simasko et al. (2002); Smith et al. (1981)
GLP‐1	Enteroendocrine L cells	GLP‐1 receptor	Activation	Suppression of food intake, enhancing insulin secretion	Iwasaki et al. (2018); Kakei et al. (2002); Krieger et al. (2016)
Peptide YY_3‐36_	Enteroendocrine L cells	Y2 receptor	Activation	Suppression of food intake	Iwasaki, Kakei, et al. (2013); Koda et al. (2005)
Short‐chain fatty acids	Produced by gut microbiota in the intestinal lumen	GPR41 (FFAR3)	Activation	Suppression of food intake	Cook et al. (2021); Goswami et al. (2018)
Bile acids	Liver	TGR5	Activation	Suppression of food intake	Wu et al. (2020)
Pancreatic hormones					
Insulin	Pancreatic β‐cells	Insulin receptor, IRS‐2	Activation	Suppression of food intake	Iwasaki, Shimomura, et al. (2013); VanderWeele (1994)
Glucagon	Pancreatic α‐cells	Glucagon receptor	Activation	Suppression of food intake	Ayush et al. (2015); Geary & Smith (1983)
Pancreatic polypeptide	Pancreatic PP‐cells	Y4 receptor	Activation	Suppression of food intake	Asakawa et al. (2003); Iwasaki, Kakei, et al. (2013)
Pituitary hormones					
Oxytocin	Posterior pituitary, along with some peripheral tissues	Oxytocin receptor	Activation	Suppression of food intake	Iwasaki et al. (2015, 2019)

*Note*: Factors that directly act on VANs in relation to changes in preference for sugar (glucose) and fat have not been elucidated. The direct action of serotonin on VANs is thought to be not strongly associated with feeding behavior (Eberle‐Wang et al., 1993).

### 3.1 Mechanical stimuli

Food intake induces distension of the stomach and intestines, and this mechanical stimulation—specifically the tension and stretch of the gastrointestinal wall—serves as a critical signal to promote meal termination (Phillips & Powley, 1996). However, it has long been recognized that gastric distension alone is insufficient to induce a true sense of satiety (Deutsch & Gonzalez, 1980; Ritter, 2004). Notably, up to 40% of ingested food may have already passed beyond the duodenum by the time individuals perceive fullness and terminate eating (Kaplan et al., 1992). Studies in humans have shown that inflating the stomach with an intragastric balloon induces only a sensation of fullness. In contrast, when nutrients such as carbohydrates or lipids are simultaneously infused into the duodenum, this gastric pressure sensation is transformed into a postprandial, meal‐like feeling of satiety (Feinle et al., 1997). Importantly, duodenal nutrient infusion without gastric distension does not elicit this meal‐like sensation (Feinle et al., 1997). These findings suggest that afferent vagal signaling evoked by gastric mechanoreceptors is integrated with nutrient‐induced afferent inputs from the intestine to generate the complex sensation of postprandial satiety.

Food intake also leads to distension of the gastrointestinal tract, and mechanical stimuli are now recognized as major activators of vagal sensory neurons. This was initially demonstrated through electrophysiological recordings of vagal afferent activity during gastric and intestinal distension (Wang et al., 2020). More recently, in vivo calcium imaging using genetically encoded calcium indicators GCaMP expressed in vagal sensory neurons has provided direct evidence that distension of the esophagus, stomach, and duodenum robustly activates vagal afferents (Lowenstein et al., 2023; Williams et al., 2016; Zhao et al., 2022).

Advances in single‐cell transcriptomics (scRNA‐seq) have enabled the characterization of gene expression profiles at single‐cell resolution, revealing remarkable cellular diversity and functional specialization across various tissues. The application of scRNA‐seq to vagal sensory neurons has uncovered the heterogeneity of nodose ganglion neurons and has begun to elucidate the molecular mechanisms underlying organ‐specific innervation. Among mechanosensitive ion channels, Piezo1 and Piezo2 have been identified as key mediators of mechanical stimuli. Both channels are expressed in subsets of vagal sensory neurons (Kupari et al., 2019), where they contribute to baroreceptor reflexes that regulate blood pressure (Zeng et al., 2018) and to mechanosensory functions in the gastrointestinal tract (Lowenstein et al., 2023). Notably, Piezo2 is expressed in vagal afferents that also express oxytocin receptors, and these neurons form IGLEs in the esophagus, stomach, and intestine, suggesting a role in gastrointestinal mechanoreception (Bai et al., 2019; Lowenstein et al., 2023; Scott et al., 2025). Furthermore, vagal afferents expressing oxytocin receptors or GLP‐1 receptors have been shown to respond to distension stimuli (Bai et al., 2019; Williams et al., 2016). However, although these peptide hormone receptors have been used as genetic markers for specific vagal neuron subtypes, their precise roles in mechanosensation and afferent activation remain incompletely understood.

Interestingly, these physiological insights are now being translated into therapeutic approaches. Hydrogel‐based ingestible capsules, which mimic the mechanical effects of a meal, have been developed as Class II medical devices approved by the U.S. Food and Drug Administration (FDA) to assist in weight management and prevent overeating (Plenity®, Epitomee®) (Aronne et al., 2022). These capsules absorb water and expand within the stomach, creating tension in the gastric wall and promoting early sensations of fullness, thereby reducing food intake. In experimental models, we have shown that administration of pectin‐containing carbonated water, which expands under acidic gastric conditions, transiently distends the stomach and intestines in mice, leading to reduced food intake and improved glucose tolerance (Ohbayashi et al., 2021). Mechanistic studies revealed that this distension stimulus increases circulating GLP‐1 levels and that GLP‐1–mediated vagal activation contributes to the regulation of feeding behavior and glucose metabolism (Ohbayashi et al., 2021). More recently, it has been reported that poorly absorbed carbohydrates can increase osmolarity in the intestinal lumen, resulting in intestinal distension and stimulating GLP‐1 secretion as a novel mechanism (Mizuma et al., 2025). Taken together, these findings highlight the pivotal role of mechanical stimulation of the stomach and intestines in modulating vagal sensory activity. This mechanosensory input, either directly or indirectly via gut hormones such as GLP‐1, is likely to regulate not only feeding behavior but also brain functions and systemic metabolism.

### 3.2 Nutrients

The mammalian body possesses highly conserved mechanisms to maintain blood glucose levels within a narrow physiological range, a process known as glucose homeostasis. Multiple organs and cell types are involved in sensing changes in blood glucose concentrations. In the hypothalamus, there are neurons that are either excited (glucose‐excited neurons) or inhibited (glucose‐inhibited neurons) in response to increases in blood glucose levels (Oomura et al., 1964, 1974). Pancreatic β‐cells in the islets also respond to rising blood glucose levels by promoting insulin secretion. Moreover, a subset of vagal sensory neurons has been shown to respond directly to glucose. Whole‐cell patch‐clamp recordings from isolated vagal sensory neurons have revealed distinct populations of neurons that undergo depolarization or hyperpolarization in response to high extracellular glucose concentrations (Grabauskas et al., 2010). The depolarization mechanism is thought to involve ATP‐sensitive potassium (K_ATP_) channels (Grabauskas et al., 2010).

While glucose can act directly on vagal sensory neurons, it also exerts indirect effects via gut‐derived signals. Luminal glucose stimulates enterochromaffin cells within the intestinal epithelium to secrete serotonin (5‐HT), which then activates vagal sensory neurons expressing 5‐HT_3_ receptors. This vago‐vagal reflex regulates gastric emptying, pancreatic exocrine secretion, and intestinal fluid secretion (Nawrot‐Porabka et al., 2013; Raybould, 2010; Raybould et al., 2003).

In appetite research, the concept of “appetition”, which refers to nutrient‐driven enhancement of feeding behavior, complements “satiation”, the process of meal termination (Sclafani, 2013). Oral glucose intake not only provides energy but also induces appetition, increasing the desire for further consumption. Notably, this glucose‐driven enhancement of appetite is not mediated by sweet taste receptors (T1R2/T1R3) in the oral cavity but instead depends on the activation of vagal afferents innervating the intestine (Tan et al., 2020). Glucose stimulates glutamatergic enteroendocrine cells that produce cholecystokinin (CCK), which are referred to as neuropod cells, through SGLT1‐mediated glucose uptake. These cells release glutamate to activate vagal afferents, thereby enhancing glucose preference and reinforcing feeding behavior (Buchanan et al., 2022).

Dietary lipids also strongly drive feeding behavior and preference. Disruption of neural pathways connecting lipid‐responsive vagal afferents to their central projection site in the NTS abolishes lipid preference (Li et al., 2022). Specific vagal sensory neurons are activated when lipids reach the intestine, a process mediated by fatty acid receptors GPR40 and GPR120. These signals are conveyed to the brain via TRPA1‐expressing vagal afferents and contribute to the formation of lipid preference (Li et al., 2022). Recent studies have further shown that vagal sensory neurons play a critical role in engaging the mesocorticolimbic dopamine system, a central reward circuit (Han et al., 2018). Remarkably, sugar and fat activate two distinct subpopulations of vagal sensory neurons, which in turn stimulate separate central reward pathways to promote dopamine release (McDougle et al., 2024). When sugar and fat are combined, dopamine release and feeding behavior are synergistically enhanced (McDougle et al., 2024). For example, pancakes with maple syrup are more likely to elicit a stronger “wanting” response when additional butter or whipped cream are added. This phenomenon may reflect the underlying neural mechanisms described above. These findings underscore the importance of nutrient‐specific activation of vagal sensory neurons in shaping food preference and appetite regulation.

### 3.3 Gastrointestinal and pancreatic hormones

Meal ingestion profoundly influences the secretion of gastrointestinal and pancreatic hormones. Vagal sensory neurons express receptors for many of these hormones and extend their nerve terminals in close proximity to enteroendocrine and pancreatic endocrine cells, allowing rapid detection of changes in hormone levels before and after meals (Table 1). Among stomach‐derived hormones, ghrelin is notable for its high circulating levels during fasting and rapid decline after food intake. Ghrelin acts to inhibit vagal afferent activity, thereby promoting feeding behavior (Yada et al., 2025). In contrast, postprandial gut hormones such as CCK, GLP‐1, and peptide YY (especially PYY_3‐36_, which is degraded by dipeptidyl peptidase‐4) activate vagal sensory neurons and induce satiation and meal termination (Iwasaki & Yada, 2012). Furthermore, pancreatic hormones, including insulin and glucagon (the latter particularly after protein‐rich meals), are also secreted postprandially and directly activate vagal sensory neurons (Ayush et al., 2015; Iwasaki, Shimomura, et al., 2013).

Among these hormones, GLP‐1 has attracted significant attention as the basis for therapies targeting type 2 diabetes and obesity. GLP‐1 secretion is stimulated by the ingestion of macronutrients such as carbohydrates, proteins, and fats. Its classical physiological role is to enhance insulin secretion, a phenomenon known as the incretin effect. However, active GLP‐1 is rapidly degraded by dipeptidyl peptidase‐4 in circulation, raising questions about whether it can reach the pancreas in sufficient amounts to exert its effects directly (Smith et al., 2014). Instead, GLP‐1 released from intestinal L‐cells may act locally on vagal sensory neurons expressing GLP‐1 receptors. This activation is then relayed to the brain, which in turn stimulates vagal efferent pathways to enhance insulin secretion from pancreatic β‐cells. This sequence of events has been termed the “neuroincretin effect” (Nakabayashi et al., 1996; Yada et al., 2025).

The vagal‐mediated effects of GLP‐1 also play a critical role in inducing satiety and preventing overeating. Our studies identified the rare sugar D‐allulose as a zero‐calorie GLP‐1 secretagogue that acutely reduces food intake and improves glucose tolerance (Iwasaki et al., 2018). These effects are abolished by subdiaphragmatic vagotomy or selective knockdown of GLP‐1 receptors in vagal sensory neurons. In a high‐fat diet (HFD) mouse model, animals exhibited hyperphagia during the light phase, leading to obesity. Daily administration of D‐allulose ameliorated this HFD‐induced hyperphagic obesity, an effect that was absent in GLP‐1 receptor knockout mice (Iwasaki et al., 2018).

Beyond macronutrients and D‐allulose, we have recently demonstrated that other rare sugars (Masuda et al., 2025), gastrointestinal distension (Ohbayashi et al., 2021), and even certain varieties of glutinous rice (Ohbayashi et al., 2024) robustly stimulate GLP‐1 secretion. These stimuli not only activate vagal sensory nerves via GLP‐1 release but also promote satiety and improve glucose tolerance. Thus, stimulating vagal sensory neurons via meal‐induced GLP‐1 secretion represents a promising non‐invasive approach to modulating feeding behavior and metabolism, distinct from vagus nerve electrical stimulation therapies that are currently under investigation.

### 3.4 Other factors

Dietary fiber is well known for its diverse health benefits, many of which are mediated by short‐chain fatty acids (SCFAs) such as acetate, propionate, and butyrate, which are produced as metabolites of the gut microbiota. SCFAs not only promote the secretion of gut hormones such as GLP‐1 and PYY but also directly activate vagal sensory neurons, thereby inducing satiation (Goswami et al., 2018). Diet composition profoundly influences the gut microbiota, which, in turn, modulates host physiology through the gut–brain axis. Emerging evidence suggests that vagal sensory pathways are involved in various microbiota‐mediated effects, such as the improvement of autism‐related behaviors and the worsening of mental functions during intestinal inflammation (Chen et al., 2025; Sgritta et al., 2019).

γ‐Aminobutyric acid (GABA), a functional amino acid found in vegetables and fermented foods, is best known as an inhibitory neurotransmitter in the central nervous system. Dietary GABA has been associated with reduced anxiety, improved cognitive function, and enhanced sleep quality in humans (Hepsomali et al., 2020). Accordingly, GABA has been incorporated into dietary supplements and functional foods in multiple countries, such as the United States and Japan. However, GABA consumed through the diet does not cross the blood–brain barrier (Kuriyama & Sze, 1971; Van Gelder & Elliott, 1958). Our recent studies revealed that oral administration of GABA alone does not activate vagal sensory neurons in mice, but when GABA is coadministered with a meal, it amplifies meal‐induced vagal activation (Nakamura et al., 2022). This enhanced vagal activation amplified the satiation induced by the meal alone (Nakamura et al., 2022). These findings raise the possibility that the brain‐related benefits of dietary GABA may, at least in part, result from its ability to potentiate meal‐induced vagal activation.

In addition, low‐osmolarity stimulation through water intake has been linked to drinking behavior via vagal sensory pathways (Ichiki et al., 2022). Intestinal perfusion of water activates a distinct population of vagal sensory neurons, which originate from the common hepatic branch of the vagus nerve. In water‐deprived mice, drinking behavior is markedly increased, and this effect is further exaggerated after surgical denervation of the common hepatic branch. Water ingestion also stimulates the secretion of vasoactive intestinal peptide (VIP), and activation of vagal sensory neurons expressing VIP receptors contributes to the suppression of excessive drinking behavior (Ichiki et al., 2022).

## 4 CLINICAL APPLICATIONS TARGETING VAGAL SENSORY NERVES

Vagotomy was originally developed as a surgical treatment for peptic ulcer disease, aiming to reduce gastric acid secretion (Dragstedt, 1969). Although temporary weight loss was observed in patients who underwent this procedure, its effects were short‐lived and the high invasiveness limited its clinical application (Ma et al., 2025).

Subsequent studies demonstrated that vagus nerve stimulation (VNS) could modulate central nervous system activity and suppress epileptic seizures in animal models (Zabara, 1992). This finding led to the development of implantable VNS therapy, in which an electrode is placed on the left cervical vagus nerve, as a novel treatment for drug‐resistant epilepsy (Sharma et al., 2025). During VNS treatment, significant weight loss was reported in approximately 62% of epilepsy patients (Burneo et al., 2002), and additional studies have suggested that VNS may prevent weight gain (Shen et al., 2025). Given these metabolic effects, VNS has also been investigated as a potential therapy for obesity. Based on preclinical and clinical findings, a modified approach known as vagal blocking therapy (VBLOC) was developed, in which electrodes are placed on the subdiaphragmatic vagus nerve and high‐frequency electrical signals are used to block vagal transmission (Camilleri et al., 2008). Clinical trials have demonstrated that VBLOC can produce moderate but significant weight loss in obese patients (Apovian et al., 2017; Ikramuddin et al., 2014).

More recently, transcutaneous auricular vagus nerve stimulation (taVNS), a noninvasive technique that does not require surgical implantation, has gained attention for its broad therapeutic potential. taVNS has been investigated for its effects on various physiological and psychological functions, including immunomodulatory and anti‐inflammatory responses, emotional recognition, attention, memory, and sleep, as well as for its potential benefits in improving anxiety and depression (Stavrakis et al., 2022; Wang et al., 2024). In animal studies, particularly in rat models, the anti‐obesity effects of taVNS have also been reported (Li et al., 2015).

## 5 CONCLUSION

Vagal sensory nerves play a critical role in relaying information about ingested food to the brain as subconscious sensory signals. These signals trigger a wide array of physiological responses. Eating is a fundamental behavior necessary for acquiring energy to sustain life. However, the rapid metabolic changes that follow food intake can also be perceived by the body as a form of stress. In this context, the ability of the brain to anticipate and respond to food‐related information through vagal sensory pathways before nutrients are absorbed into the systemic circulation may represent an essential mechanism for maintaining homeostasis. Moreover, understanding this vagal pathway holds potential for developing novel therapeutic strategies for metabolic disorders such as obesity and diabetes, as well as for improving mental health conditions including depression.

## AUTHOR CONTRIBUTIONS

K.I. and Y.I. conceived the idea for the manuscript, K.I., R.K., and Y.I. wrote and edited the text, and prepared the figures and tables. All authors contributed to the revision of the manuscript.

## FUNDING INFORMATION

No funding information provided.

## CONFLICT OF INTEREST STATEMENT

The authors declare no conflict of interest.

## ETHICS STATEMENT

Not applicable.
